# Trogocytosis with monocytes associated with increased α2,3 sialic acid expression on B cells during H5N1 influenza virus infection

**DOI:** 10.1371/journal.pone.0239488

**Published:** 2020-09-18

**Authors:** Supasek Kongsomros, Maytawan Thanunchai, Suwimon Manopwisedjaroen, Prasit Na-Ek, Sheng-Fan Wang, Tana Taechalertpaisarn, Arunee Thitithanyanont

**Affiliations:** 1 Department of Microbiology, Faculty of Science, Mahidol University, Bangkok, Thailand; 2 Department of Clinical Pathology, Faculty of Medicine, Vajira Hospital, Navamindradhiraj University, Bangkok, Thailand; 3 School of Medicine, Walailak University, Thasala, Nakhon Si Thammarat, Thailand; 4 Department of Medical Laboratory Sciences and Biotechnology, College of Health Sciences, Kaohsiung Medical University, Kaohsiung City, Taiwan; Erasmus University Medical Center, NETHERLANDS

## Abstract

The immunopathogenesis of H5N1 virus has been studied intensively since it caused cross-species infection and induced high mortality to human. We previously observed the interaction between monocytes and B cells, which increased the susceptibility of B cell to H5N1 virus infection after a co-culture. Levels of α2,3 sialic acid (avian flu receptor) were also significantly increased on B cell surface in this co-culture model with unclear explanation. In this study, we aimed to determine the possible mechanism that responded for this increase in α2,3 sialic acid on B cells. Acquisition of α2,3 SA by B cells via cell contact-dependent trogocytosis was proposed. Results showed that the lack of α2,3 SA was detected on B cell surface, and B cells acquired membrane-bound α2,3 SA molecules from monocytes in H5N1-infected co-cultures. Occurrence of membrane exchange mainly relied on H5N1 infection and cell-cell contact as opposed to a mock infection and transwell. The increase in α2,3 SA on B cell surface mediated by trogocytosis was associated with the enhanced susceptibility to H5N1 infection. These observations thus provide the evidence that H5N1 influenza virus may utilize trogocytosis to expand its cell tropism and spread to immune cells despite the lack of avian flu receptor.

## Introduction

Avian influenza H5N1virus has caused outbreaks in domestic poultry and in humans, with a mortality rate exceeding 50% in infected humans. Although there has been no new human cases of H5N1 infection as of 2018 [1], the virus has been continuously circulating in wild birds and waterfowl. This virus has the potential of generating new reassortant strains and may acquire the ability to transmit from human to human, posing a global pandemic threat. Thus, a clearer understanding of the pathogenesis of previously emergent H5N1 viruses is critical for preparing for this looming threat.

A number of studies regarding H5N1 pathogenesis have explored tissue tropism and host immune responses towards the virus in order to explain the systemic spread and cytokine storm observed in H5N1 patients [2–4]. These studies indicate that the important factor for determination of H5N1 viral tropism is sialic acid (SA) [5–7]. Avian influenza virus preferentially recognizes the α2,3-linked sialic acid receptor (α2,3 SA), while human influenza virus preferentially binds the α2,6-linked sialic acid receptor (α2,6 SA). α2,3 SA-expressing cells are predominant and permissive to H5N1 infection, whereas the cells or tissues that lacked α2,3 SA were resistant to H5N1 infection [8, 9]. For example, ciliated epithelial cells distributed throughout the human upper respiratory tract are unable to be recognized by the H5N1 virus due to low abundance of α2,3 SA, resulting in the limitation of H5N1 replication in the human upper respiratory tract [9]. H5N1 virus has been reported for its broad tissue tropism as well as the ability to infect several cell types with high replication rates. Furthermore, H5N1 was also reported to induce a robust host response in multiple organs according to the case reports and *in vitro* studies [2, 3, 10, 11]. During viremia, H5N1 directly encounters peripheral blood immune cells, and thus peripheral blood is primarily used for *in vitro* H5N1 evaluation. Several reports indicate that H5N1 can infect human peripheral blood mononuclear cells (PBMCs) including monocytes, natural killer (NK) cells as well as B and T lymphocytes, resulting in impaired cytokine production, cell activity and functionality *in vitro* [12–15]. In addition, viral RNA and protein of H5N1 were recovered from PBMCs of fatal cases, indicating that PBMC can carry the virus and help the H5N1 virus spread to other tissues [16].

Our previous studies demonstrated the susceptibility of PBMCs to H5N1 infection and demonstrated the role of PBMCs in H5N1 pathogenesis [14, 17]. We found an increase in susceptibility of B cells to infection upon the increase in the α2,3 SA receptor during a direct contact with monocytes. However, the mechanism responsible for the increased level of α2,3 SA on the B cell surface has not been completely understood. The interaction between two distinct immune cells can lead to the formation of an immunological synapse where membrane exchange can occur. This phenomenon is termed trogocytosis [18]. Trogocytosis is a ubiquitous process predominantly documented in immune cells such as T cells, B cells, and dendritic cells (DCs) [19–21]. It typically involves the transfer of membrane-anchored antigens from donor cells to recipient cells, thereby promoting immune responses by enhancing antigen presentation, cell proliferation, and sustained intracellular signaling. However, trogocytosis can also lead to the detrimental membrane transfer of viral receptors and virus particles from infected cells, resulting in altered permissiveness of recipient cells to the virus and facilitation of the spread of virus, respectively. Previously reported evidences included the case that NK cells acquired a receptor for Epstein-Barr virus (EBV) from EBV-infected B cells, enabling the EBV binding onto NK cells [22], Another example was dendritic cell acquisition of HIV-1 from infected T cells via an antigen uptake mechanism through cell-cell contact, proposed as a mechanism in which HIV-1 exploits the immunological synapse to disseminate virus to less accessible organs [23].

In present study, our results demonstrated that B cells acquired membrane-bound α2,3 SA molecules from monocytes via trogocytosis triggered by the H5N1 virus. The presence of monocyte-derived α2,3 SA receptors could subsequently enable the H5N1 virus to infect B cells. Hence, we propose that trogocytosis with monocytes might be a potential mechanism responsible for the increased level of α2,3 on the B cell surface, which could explain the enhanced susceptibility of B cells to H5N1 infection.

## Materials and methods

### B cell and monocyte isolation

Blood samples were collected from healthy donors with informed consent approved by the ethical clearance committee on human rights related to research involving human subjects (protocol number 03-50-25) at Faculty of Medicine Ramathibodi hospital, Mahidol University, Thailand. Peripheral mononuclear cells (PBMCs) were isolated from blood samples by density gradient centrifugation using Lymphoprep TM (Axis-Shield, Oslo, Norway). B cells and monocytes were isolated from PBMCs using microbeads conjugated to monoclonal anti-human CD20 and CD14 antibodies, respectively, by MACS^®^ cell separation technology (Miltenyi Biotec, Germany) following the manufacturer's instructions. Isolated B cells and monocytes were maintained in RPMI 1640 (Gibco, NY, USA) supplemented with 10% FBS (Gibco, NY, USA), 100 U/ml penicillin and 100 μg/ml streptomycin. Flow cytometry was performed to assure purity of B cells and monocytes by staining with anti-human CD20-PE (BD biosciences, CA, USA) and anti-human CD14 APC-conjugated (Immunotools, Friesoythe, Germany), respectively. More than 95% purity was acceptable for further experiments.

### Virus

A highly pathogenic avian influenza (HPAI) H5N1 virus strain A/open-billed stork/Nakhonsawan/BBD0104F/04 was isolated from a cloacal swab of an Asian open-billed stork. Viral stocks were propagated in MDCK cells. Briefly, a MDCK monolayer was inoculated with influenza virus. After 1 hour of adsorption, all medium was removed and replaced with fresh culture medium: Minimum Essential Medium (MEM) (Gibco, NY, USA) with 100U/ml penicillin and 100 μg/ml streptomycin. Infected cells were incubated at 37°C with 5% CO_2_. Supernatant was harvested when a 3+ to 4+ cytopathic effect (CPE) was observed. Viral titers were determined by plaque assay. All procedures involving H5N1 virus were performed in a certified Biosafety level 3 laboratory at the Department of Microbiology, Faculty of Science, Mahidol University, Thailand.

### H5N1 infection of B cells and monocytes

Primary B cells and monocytes at cell densities of 10^6^ cells/mL were inoculated with H5N1 virus at a multiplicity of infection (MOI) of 1 for 1 hour. Following adsorption, cells were washed, and the medium was replaced with fresh RPMI 1640 supplemented with 10% FBS, 100 U/ml penicillin and 100 μg/ml streptomycin.

### Co-culture and transwell assay

For co-culture assays, infected B cells and infected monocytes (2×10^5^ cells) were added into a 96-well plate at a ratio of 1:1 and cultured for 0, 12 and 24 hours. For transwell assays, infected B cells (3×10^5^ cells) were added onto a 0.4 μm polycarbonate transwell insert (Corning, NY, USA). Infected monocytes (3×10^5^ cells) were then added in the lower chamber at a ratio of 1:1 and cultured for 0, 12 and 24 hours. In parallel, negative control infection cultures (mock) were performed using uninfected B cells and monocytes in both co-culture and transwell assays. In some experiment, monocytes were labeled with the fluorescent cell linker PKH26 Red (Sigma-Aldrich, USA) and subsequently infected with H5N1 virus. Infected PKH26-labeled monocytes (2×10^5^ cells) were co-cultured with infected B cells (2×10^5^ cells) for 0, 12 and 24 hours. At the end of the culture periods indicated, the cells were harvested by vigorous pipetting to generate a homogenous suspension and subjected to flow cytometry and immunofluorescence microscopy.

### Flow cytometry

Cells were washed with 0.1% BSA in PBS, followed by specific surface staining with 1:50 APC-conjugated anti-CD14 (immunotools, Friesoythe, Germany) and 1:20 PE-conjugated anti-CD20 (BD biosciences, CA, USA) at 4°C for 30 min. Cells were then washed and fixed with 4% paraformaldehyde at 4°C for 30 min. Subsequently, cells were stained for surface α2,3 SA with 1:100 biotinylated Maackia Amurensis Lectin I (MAL I) (Vector Laboratories, CA, USA) at 4°C for 30 min. After washing, cells were incubated with 1:200 Per-CP Streptavidin (BD Biosciences, CA, USA). For intracellular staining of viral NP, cells were permeabilized in Cytofix/Cytoperm reagent (BD Biosciences, CA, USA) at 4°C for 10 min, then washed with Perm/wash (BD Biosciences, CA, USA), incubated with 1:500 FITC-conjugated anti-NP antibody (Millipore, USA) at 4°C for 30 min, washed and suspended in 2% paraformaldehyde. To detect α2,3 SA expression for single stain, cells were stained with 1:100 FITC-conjugated MAL I (Vector Laboratories, CA, USA). Stained cells were detected using a CytoFLEX flow cytometry (Beckman Coulter, USA), and the data were analyzed using Kaluza Analysis Software—Version 2.0. (Beckman Coulter, USA). A total of 10,000 cells were acquired for each flow cytometry analysis.

### Immunofluorescence microscopic imaging

Cellular membranes of B cells were labeled with the fluorescent cell linker PKH26 Red (Sigma-Aldrich, USA) following the manufacturer's instructions before co-culture. PKH26 labeled B cells and monocytes were adsorbed with H5N1 virus at an MOI of 1 for 1 hour. Mock infection controls were performed in parallel. B cells and monocytes (3×10^5^ cells) were co-cultured at a ratio of 1:1. After 12 hours of co-culture, the cells were collected and suspended in PBS. 10 μL the cell suspension was dropped onto superfrost Plus slides (Erie Scientific, NH, USA). After air-drying, the cells were fixed with 4% paraformaldehyde, rinsed with PBS and blocked with 1% BSA in PBS at room temperature for 1 hour. To detect α2,3 SA and CD14 expression on the cell surface, the cells were stained with 1:50 FITC-conjugated Maackia Amurensis Lectin I (MAL I) (Vector Laboratories, CA, USA) and 1:25 APC-conjugated anti-CD14 (immunotools, Friesoythe, Germany) at room temperature for 1 hour. Cells were rinsed with PBS, followed by deionized water, and then mounted in Prolong Gold antifade reagent containing DAPI counter stain (Invitrogen, CA, USA). Immunostained cells were analyzed by laser confocal microscopy (Olympus FV 1000)

### Data analysis

All data are representative of three independent experiments. The values are expressed as mean ± SEM. Statistical analysis was performed using two-way ANOVA. *P < 0.05, **P < 0.01, ***P < 0.001. The correlation was performed using Pearson correlation.

## Results

### 1. High expression of α2,3 SA is detected on monocytes, but not on B cells

**Fig 1:**
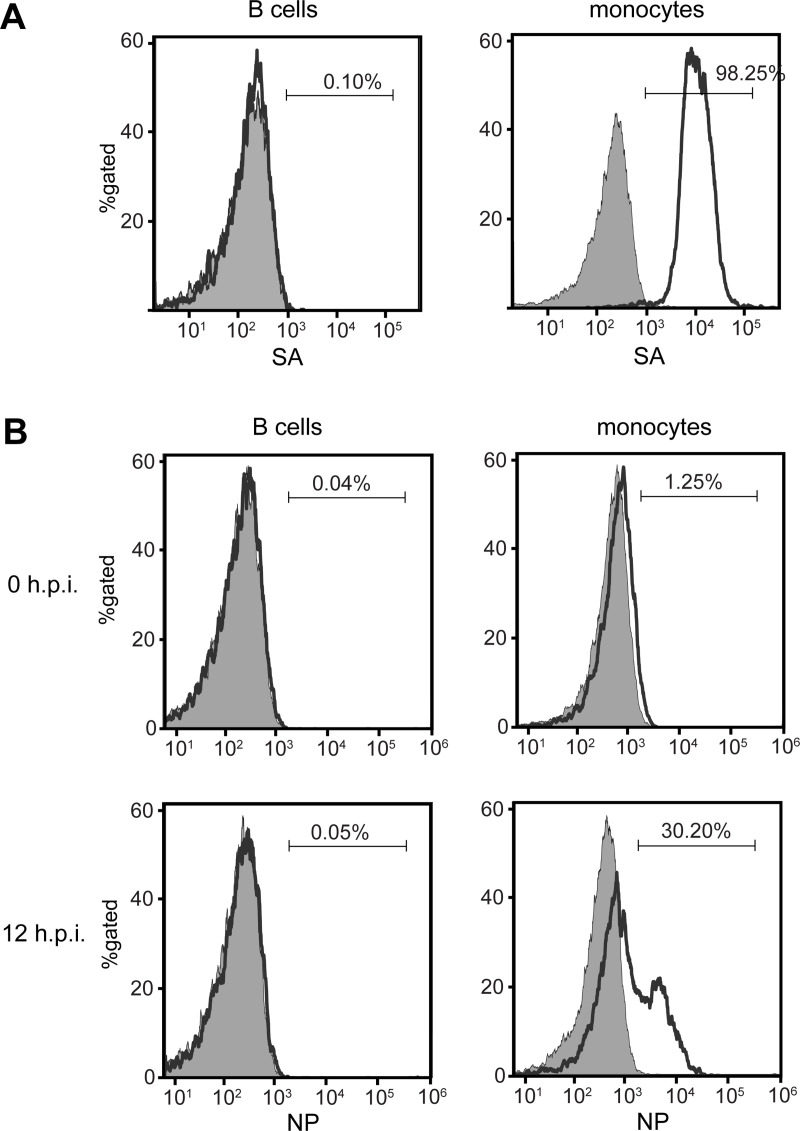
High expression of α2,3 SA is detected on monocyte but not on B cell surface. Sialic acid receptors were detected on isolated B cells and monocytes by lectin staining with MAL I. B-cells and monocytes are identified by gating the CD20- and CD14-positive cells, respectively. (A) Flow cytometry analysis shows the percentage of B cells (left panel) and monocytes (right panel) positive for surface α2,3 SA (open histograms) relative to the unstained cells (grey histogram). B cells and monocytes were infected with H5N1 virus at MOI of 1 for 12 hours and subjected to flow cytometry after NP staining. (B) NP expression on B cells and monocytes at 0 and 12 hours post infection (h.p.i.) are shown (H5N1 infection, open histogram; mock infection, grey histogram). Histograms are representative of three independent experiments.

According to our previous results, purified monocytes were susceptible to H5N1 infection, whereas purified B cells were shown resistant. We suspected that this resistance on B cells to H5N1 infection was correlated with the levels of surface α2,3 SA expression. The human primary B cells and monocytes were purified from healthy donors using anti-CD20 and anti-CD14 antibodies coated magnetic beads, and then stained with FITC-conjugated MAL I (a lectin with high affinity to α2,3 SA molecules) and subjected to flow cytometry. Our results showed that there was no difference in the α2,3 SA expression on B cells compared to the unstained control cells (Fig 1A, left panel). Approximately 98% of monocytes were found to express α2,3 SA as expected (Fig 1A, right panel). To assess the degree of infection, the human primary B cells and monocytes were infected with H5N1 virus at MOI of 1 for 12 hours. Infected cells were subjected to flow cytometry after viral nucleoprotein (NP) staining. We found that approximately 30% of monocytes were positive for NP expression, whereas in B cells, NP expression was not detected compared to mock controls and 0 hour post infection (h.p.i.) (Fig 1B). Our results indicate that α2,3 SA expressing levels on primary monocytes and B cells are correlated with the degree of H5N1 infectivity.

### 2. H5N1 infection and cell-cell contact are essential for the increased expression of α2,3 SA receptor on B cells

**Fig 2:**
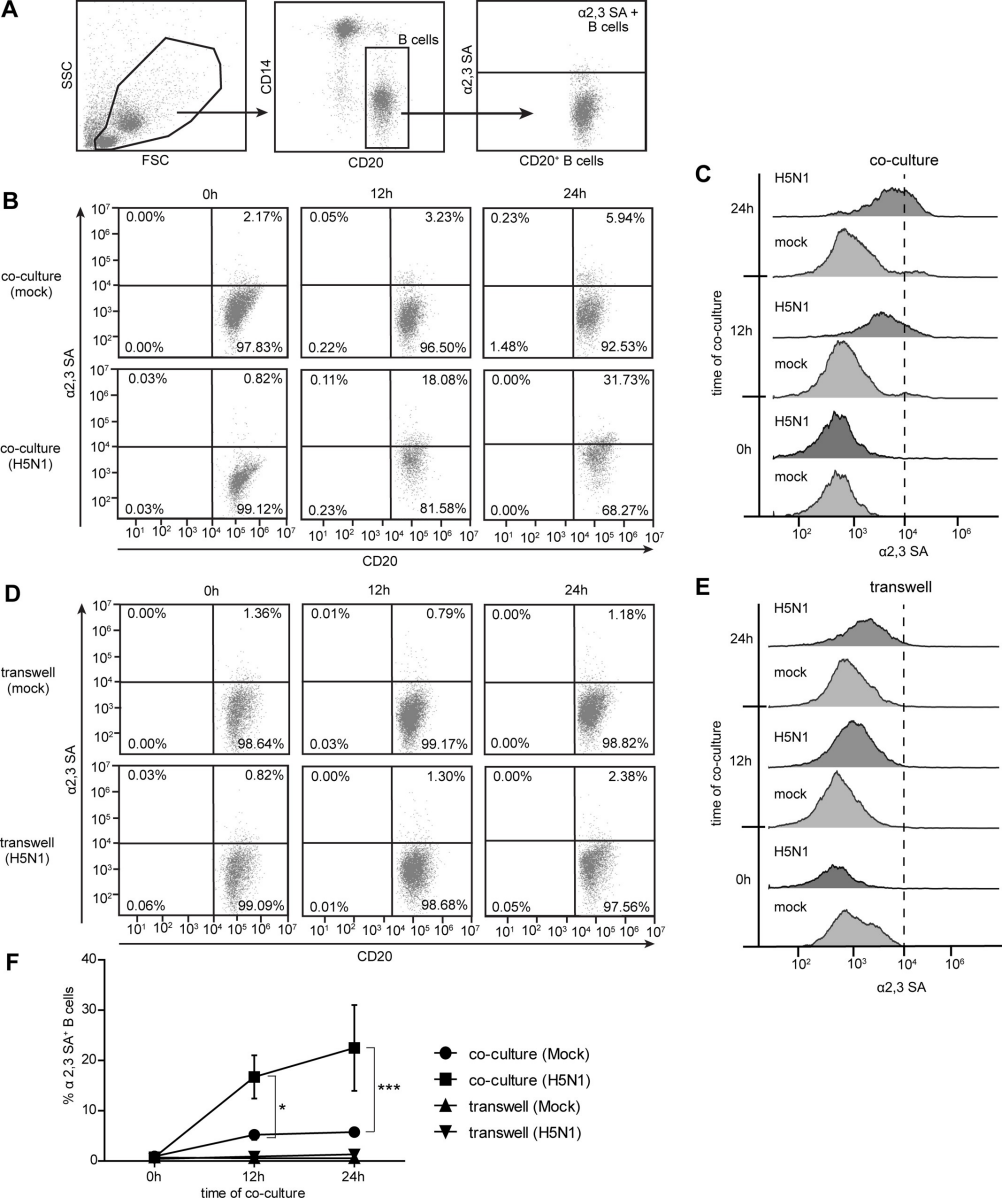
H5N1 virus and cell-cell contact are required for increased expression of α2,3 SA on B cells. H5N1 infected monocytes and B cells were co-cultured or indirect cultured in a transwell for 0, 12 and 24 hours. Mock infection controls were performed in parallel. The cells were then harvested, and CD20 (B cell marker) and α2,3 SA staining was performed, and the cells were analyzed by flow cytometry. (A) CD20^+^ B cells were gated to identify α2,3 SA expression. The kinetics of α2,3 SA expression on B cells in direct co-cultures and transwells are shown in (B) and **(**D**)**, respectively, *(Mock infection*, *upper panel; H5N1 infection*, *lower panel)*. The MFI histograms of α2,3 SA expression on B cells are demonstrated in (C) and (E). Percentages of α2,3 SA^+^ B cells are summarized in (F). The data represent as mean ± SEM from three independent experiments (three donors). Statistical analysis was performed using two-way ANOVA. *P < 0.05, ***P < 0.001.

We previously proposed that α2,3 SA on B cells was up-regulated after a co-culture with monocytes, contributing to higher susceptibility of B cells to H5N1 infection. In this study we examined the kinetics of α2,3 SA expressions on B cells. Human primary B cells and monocytes were purified. Primary B cells were co-cultured with monocytes via direct and indirect contact co-culture (transwell) with/without H5N1 infection for 0, 12, and 24 hours. After MAL I staining, the co-cultures were subjected to flow cytometry (S1 Fig). The CD20^+^ as B cell marker was used to gate B cells and identified α2,3 SA expression (Fig 2A). We observed a time-dependent increase in α2,3 SA on B cells via direct cell-cell contact with monocytes in H5N1 infection compared to that in mock infection (Fig 2B and 2C). However, we did not detect any significant increase in α2,3 SA on B cells in transwells, even though the B cells were indirectly co-cultured with monocytes in H5N1 and mock infection (Fig 2D and 2E). The results summarized in Fig 2F indicated that α2,3 SA expression significantly increased on B cells in the direct co-cultured with monocytes upon H5N1 infection at 12 and 24 hour of co-cultures (0.76±0.20%, 16.72±4.29%, 22.48±8.54% at 0, 12, 24 hours, respectively). These results suggest that H5N1 infection and cell-cell contact are crucial for increased expression of the α2,3 SA receptor on B cells.

### 3. B cells acquire α2,3 SA receptor from monocytes via trogocytosis

**Fig 3:**
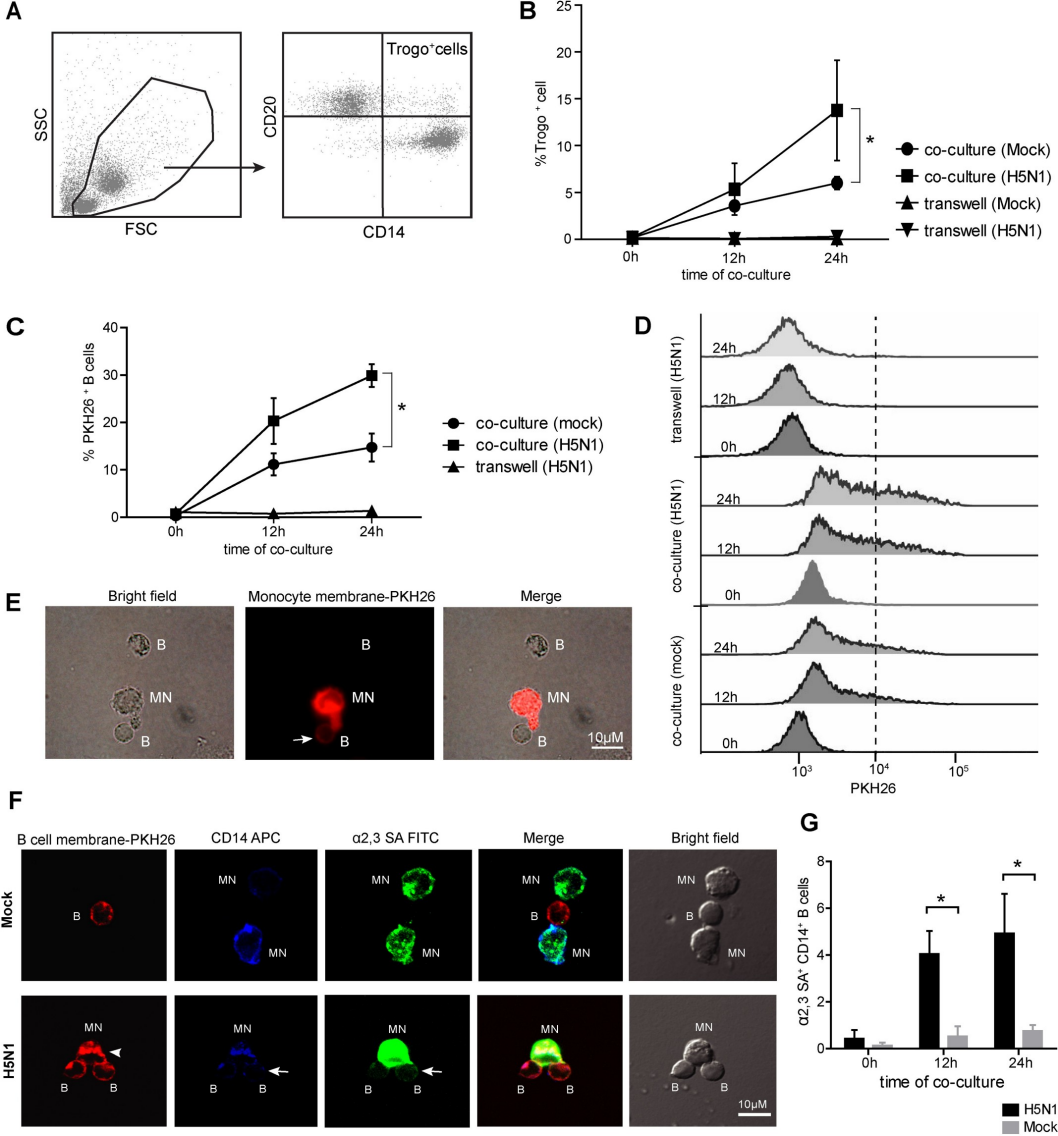
B cells acquire membrane-bound α2,3 SA from monocytes through trogocytosis upon activation by H5N1 infection. H5N1 infected monocytes and B cells were co-cultured or cultured separately in transwell for 0, 12 and 24 hours. Mock infected cultures were performed as controls. The cells were then harvested, and CD20 (B cell marker) and CD14 (monocyte marker) staining was performed, and the cells were analyzed by flow cytometry. (A) CD20^+^CD14^+^ expressing cells were gated to identify trogocytosis prositive cells (Trogo^+^ cells). Kinetics of the increase in Trogo^+^ cells in direct contact co-cultures and transwells are shown in (B). The H5N1 infected PKH26 pre-labeled monocytes and B cells were co-cultured or cultured separately in transwell for 0, 12 and 24 hours. Mock infected cultures were performed as controls. The cells were then harvested and analyzed by flow cytometry. Only B cells (CD14 negative cells) were gated to identify PKH26 expression. Kinetics of the increase in PKH26^+^ B cells are summarized in (C). The MFI histograms of PKH26 expression on B cells are demonstrated in (D). (E) Representative fluorescent microscopy images show that the plasma membrane of monocyte was transferred to B cell in a cell-contact dependent trogocytosis. (F) Representative microscopy images of co-culture of B cells and monocytes with/without H5N1infection taken by laser confocal microscopy show that infected B cells acquire the CD14 monocyte marker and α2,3 SA (arrow) in contact with infected monocytes (lower panel), whereas B cells in contact with uninfected monocyte do not (upper panel). B cell membrane is also found in monocytes (arrowhead) indicates the bidirectional directional membrane transfer between those cells. The transfer of monocyte-derived α2,3 SA receptors to B cells was analyzed by flow cytometry. B cells were gated to identify α2,3 SA and CD14 monocyte marker expression. The percentage of α2,3 SA^+^ CD14^+^ B cells in H5N1 infected and mock are shown (G). The data represent as mean ± SEM from three independent experiments (three donors). Statistical analysis was performed using two-way ANOVA. *P < 0.05, ***P < 0.001. Scale bare-10 μm.

Trogocytosis is a biological phenomenon by which cells share membrane and membrane-associated proteins during cell-cell conjugation. The result in Fig 1 showed that B cells did not express α2,3 SA receptor, and α2,3 SA molecules detected on B cells were possibly donated from other cells through cell-cell contact. We, therefore, suspected that B cells might acquire α2,3 SA through trogocytosis from monocytes. To demonstrate that H5N1 infection involved in transferred membrane between the two cells, the purified primary B cells and primary monocytes were obtained using anti-CD20 and anti-CD14 beads and then subjected to direct or indirect co-culture (transwell) for 0, 12, and 24 hours, with/without H5N1 infection. The cells were subjected to flow cytometry analysis after APC-conjugated anti-CD14 and PE-conjugated anti-CD20 staining (S1 Fig). The CD20^+^ and CD14^+^ markers were gated to identify trogocytosis-positive cells (Trogo^+^ cells) (Fig 3A). Further, our data indicated that percentages of Trogo^+^ cells were significantly increased in a time-dependent manner in H5N1 infected co-cultures compared to those in mock infected co-cultures (p<0.05). On the other hand, Trogo^+^ cells were not detected in indirect transwells (Fig 3B). Notably, small amounts of Trogo^+^ cells were detected in the mock infected co-cultures, indicating that the membrane exchange may occur when two different cells were in direct contact (Fig 3B). To further confirm if (i) B cells could obtain the plasma membrane fragment from monocytes via trogocytosis and (ii) H5N1 infection may trigger trogocytosis, the PKH26 fluorescent tracer pre-labeled purified monocytes were co-cultured with purified B cells for 0, 12 and 24 hours, with or without H5N1 infection. The expression of PKH26 was analyzed by flow cytometry (S2 Fig). Our results revealed that the percentages of PKH26^+^ CD20^+^ primary B cells were significantly higher in H5N1 infected co-cultures compared to those in mock infected co-cultures, whereas PKH26^+^CD20^+^ primary B cells were not detected in all transwells (Fig 3C and 3D). However, the expressions of PKH26 on primary B cells were slightly increased in the direct co-cultures under mock infection (Fig 3C and 3D). Similar results were found in fluorescence microscope observation, indicating that PKH26 labeled monocytic plasma membranes were transferred to B cell via direct contact (Fig 3E). To assure that B cells acquired the membrane-bound α2,3 SA receptor from monocytes via trogocytosis, the PKH26-labeled primary B cells were co-cultured with primary monocytes, with or without H5N1 infection. After 12 hours of co-culture, the cells were stained with FITC-conjugated MAL-I and APC-conjugated anti-CD14, and then subjected to confocal microscope analysis (S3 Fig). Results showed that H5N1 infection enhanced PKH26-labeled B cell membrane transfer to APC-labeled monocytes and vice versa (Fig 3F). In addition, H5N1 infection also triggered MAL-I stained α2,3 SA receptors transfer from monocytes to B cells (Fig 3F). These phenomena were not observed in mock infection control (Fig 3F). The transfer of monocyte-derived α2,3 SA receptors to B cells was quantified by flow cytometry by detecting the expression of α2,3 SA and CD14 monocyte marker on B cells. We found that the percentages of α2,3 SA^+^ CD14^+^ B cells in the H5N1 infection were 0.430±0.36%, 4.04±0.99% and 4.92±1.68% at 0, 12, 24 hours, respectively, and were significantly higher than in the mock infection (0.13±0.13%, 0.52±0.438 and 0.76±.26% at 0, 12, 24 hours, respectively) (Fig 3G). These findings confirm that B cells acquire the membrane-bound α2,3 SA receptors from monocytes via trogocytosis upon activation by the H5N1 virus.

### 4. The acquisition of α2,3 SA on B cell surfaces by trogocytosis is associated with the enhancement of susceptibility to H5N1 infection

**Fig 4:**
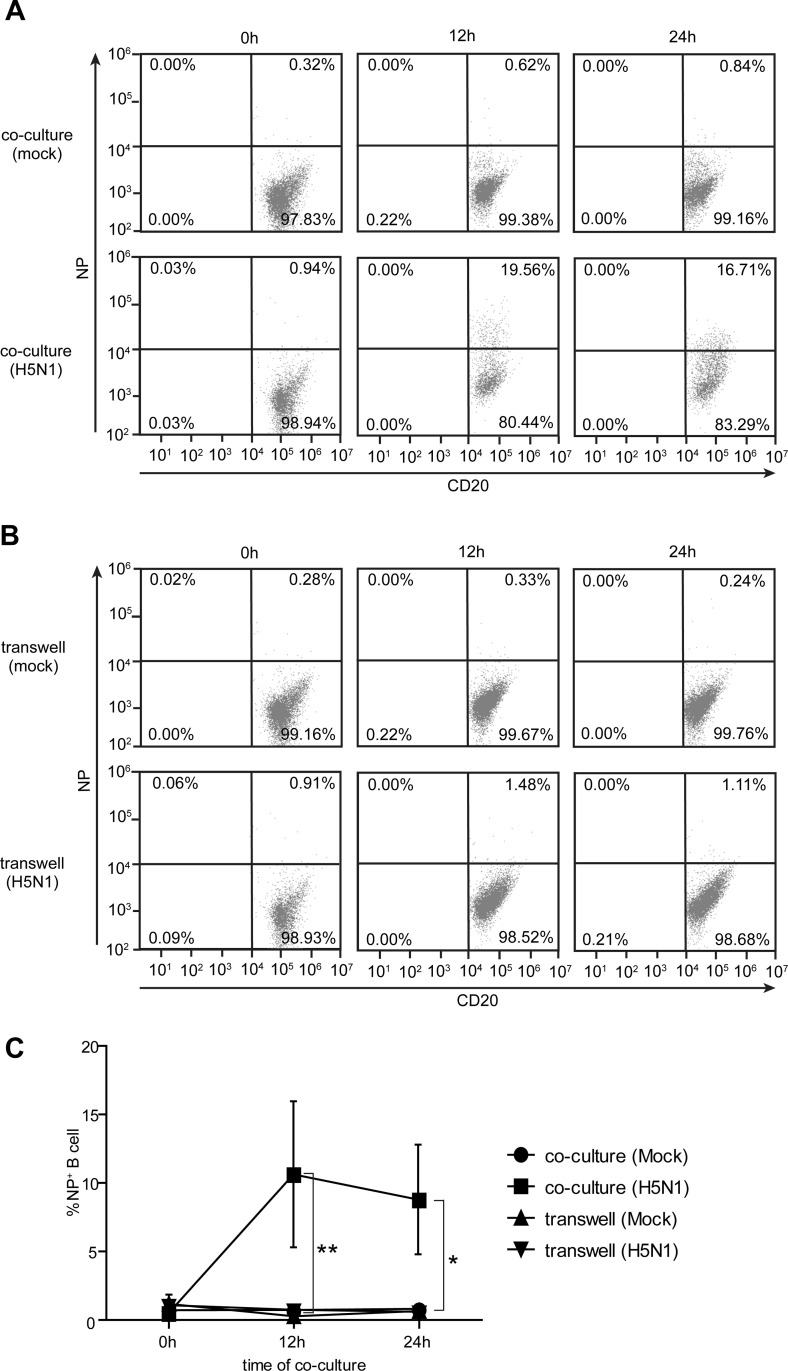
H5N1 infection is increased on B cell after direct contact co-culture with monocytes. Detection of influenza viral NP expressed on B cells after co-culture and culture in transwell with monocytes for 0, 12 and 24 hours are shown in (A) and (B), respectively, *(Mock infection*, *upper panel; H5N1 infection*, *lower panel)*. Percentages of NP^+^ B cells are summarized in (C). The data represent as mean ± SEM from three independent experiments (three donors). Statistical analysis was performed using two-way ANOVA. *P < 0.05, **P < 0.01.

**Fig 5:**
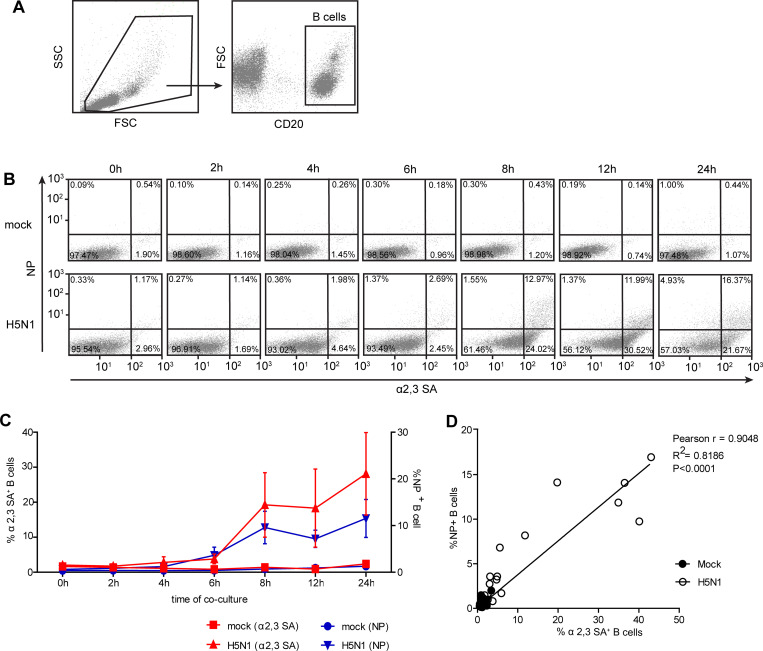
The acquisition of α2,3 SA on B cell surfaces by trogocytosis is associated with enhanced susceptibility to H5N1 infection. B cells and monocytes were co-cultured with/without H5N1 infection for 0, 2, 4, 6, 8, 12, and 24 hours. CD20, α2,3 SA and influenza viral NP staining was performed, and the cells were analyzed by flow cytometry. (A) CD20^+^ B cells were gated to identify α2,3 SA and NP expression. (B) α2,3 SA and NP expression on B cells in H5N1 infected and mock are shown *(Mock infection*, *upper panel; H5N1 infection*, *lower panel)*. Percentages of α2,3 SA^+^ (left Y-axis) and NP^+^ B cells (right Y-axis) are summarized in (C) (mean ± SEM; n = 3). (D) The correlation (Pearson correlation) between α2 SA^+^ and NP^+^ on B cells is shown. The line represents to the linear regression results; Pearson r correlation coefficient, R2 coefficient of determination and p-value are shown in the right top.

It was hypothesized that B cells acquired membrane-bound α2,3 SA receptors from monocyte during co-cultures, leading to the enhancement of their susceptibility to H5N1 infection. To verify that hypothesis, the co-culture and transwell assays using primary B cells and monocytes with/without H5N1 infection (S1 Fig) were performed. The cells were stained with anti-NP and anti-CD20, and then subjected to flow cytometry. Results showed that NP^+^ B cells were significantly increased in direct contact with monocytes in H5N1 infected co-cultures at 12 and 24 hours compared to those in transwells (p<0.05) (Fig 4), indicating that the interaction with monocytes resulted in the enhanced susceptibility of B cells to H5N1 infection. Next, we evaluated the correlation between the presence of α2,3 SA receptors on B cell and H5N1 infection. The CD20^+^ B cells were gated to identify α2,3 SA and NP expression (Fig 5A). Flow cytometry analysis showed that a gradual increase in α2,3 SA and NP expression on B cells were observed in H5N1 infected co-cultures at different time points but not in mock infected co-cultures (Fig 5B and 5C). Higher α2,3 SA^+^ NP^+^ B cells were observed after co-culture for at least 8 hours (Fig 5C). In addition, a strong positive correlation (Pearson r = 0.9048; P <0.0001) between α2,3 SA receptor expression and H5N1 infection on B cells was observed (Fig 5D). These data suggest that the α2,3 SA acquisition of B cells by trogocytosis with monocytes is associated with their susceptibility to H5N1 infection.

## Discussion

Highly pathogenic influenza H5N1 virus causes severe diseases in multiple organs and is characterized by an approximately 60% mortality rate. Viral pathogenesis is associated with broad cell and tissue tropisms, especially in immune cells, leading to a robust host immune response with induction of aberrant levels of cytokine production. A number of published studies reported the high susceptibility of immune cells such as macrophages, monocytes, dendritic cells, B cells, and T cells to H5N1 infection. The outcomes of viral infection include dysregulated cytokine production and poor cell viability [12, 13, 24, 25]. In this study, we were interested in exploring the interplay between the H5N1 virus and immune cells purified from PBMCs since this situation mimics the condition during viremia. Our previous data demonstrated an unexplained interaction between primary B cells and monocytes which promoted α2,3 SA expression on the B cells and was associated with enhanced susceptibility of the B cells to H5N1 infection [14]. However, the mechanism of the increase in α2,3 SA on B cells remained undefined. In this study, flow cytometry and confocal microscopy results revealed the membrane transfer between B cells and monocytes during co-culture. The results clearly demonstrated that membrane-bound α2,3 SA molecules were transferred from monocytes to the B cell surfaces which were associated with the increased susceptibility of the B cells to H5N1 infection. Similar previously published studies by Tabiasco J. *et al* and Aucher A. *et al* supported our data, demonstrating that NK cells and CD8^+^ T cells acquired the virus receptors from their target cells and expressed these receptors in the correct orientation on their membranes. This subsequently allowed the virus to bind to novel targets [22, 26]. It was believed that this transfer of virus receptors to another cell type might be a strategy employed by the virus to establish infection in receptor-lacking cells and disseminate the virus to less accessible tissues.

The kinetics of the trogocytosis demonstrated the role of the H5N1 virus as a stimulus for trogocytosis because the membrane exchange was distinctly observed at the time of completion of the first viral replication cycle (8 hours) (Fig 5C). These attempts to establish infection of B lymphocytes might be one of the mechanisms the H5N1 virus employed to promote its dissemination to peripheral lymphoid organs and other organs. This phenomenon is relevant to *in vivo* situations in which various immune cells are in contact with each other upon being exposed to the H5N1 virus. Following trogocytosis, B cells transiently expressed α2,3 SA receptors and were readily infected by the H5N1 virus. Moreover, the B cells could possibly carry the H5N1 virus while migrating from the blood stream to the lymph nodes, acting as a ‘Trojan Horse’ to carry in virus undetected.

Trogocytosis involves the transfer of entire membrane patches. In previous study of *Francisella tularensis* infection, trogocytosis was increased in both primary cells and mouse model experiments revealing that a bacterial stimulus increased the rate of trogocytosis [27]. Little is known regarding virus-mediated trogocytosis in B cells. Although pathogen transfer-mediated trogocytosis has not been reported in B lymphocytes, this phenomenon was evidenced in DCs which acquired HIV-1 particles from infected T cells in a contact dependent manner. This was believed to be another efficient mode for HIV transmission and dissemination [23]. Our presented data demonstrated that the H5N1 virus appeared to drive the membrane exchange between B cells and infected monocytes. Previously, there were reported that B cells can recognize and acquire the membrane-bound antigen through the caption of plasma membrane from the antigen presenting cells (APCs) [19, 28]. The interaction of the B cell receptor (BCR) and antigen tethered at the APC surfaces was identified as a potential trigger of trogocytosis on B cells [29]. Induction of trogocytosis after H5N1 infection suggested that interaction of the BCR with the viral antigens at the surface of infected monocytes may initiate and enhance the trogocytosis. Additionally, the BCR also acts as a signaling receptor, thus the binding of antigen to the BCR introduces B cell to activate [30]. Thus, the transfer of virus particles aggregated in the plasma membrane of infected monocytes to B cells is highly possible as B cells are antigen presenting cells with the ability to internalize cell-associated antigen for further processing and presentation of antigens to effector T cells [19, 31]. Whether H5N1 particles on membrane patches transferred to B cells during their conjugation with monocytes need to be further investigated. There has been reported that trogocytosis can be either unidirectional or bidirectional transfer [32]. Our previous studies demonstrated the massive cell death of monocytes after 9-hour of H5N1 infection but not B cells (data not shown). Thus, in this study the major population of Trogo^+^ cells at 24-hour post infection in Fig 3B were likely of B cell origin and plasma membrane of monocytes was likely transferred to B cells instead of vice-versa. Whether B cells mediated trogocytosis leads to death of monocytes need to be further explored. Further, the fact that blocking of trogocytosis could inhibit α2,3 SA expression as well as H5N1 infection may be of important for development of therapeutics. Future work will be required in order to confirm that the H5N1 virus utilizes the mode of cell-to-cell transmission as an alternative mechanism for systemic spread and the exact role of H5N1 infection driving trogocytosis is needed to be further investigated.

In conclusion, our results demonstrate that B cells acquire the α2,3 SA receptors from monocytes through contact-dependent trogocytosis. The membrane exchange during the interaction of B cells and monocytes was evidently induced by the H5N1 virus and led to the increase in α2,3 SA receptors on B cells. The ability of the H5N1 virus to induce trogocytosis might be considered as a potential mechanism for establishing infection in receptor-lacking cells, especially cells of the immune system. This study provides better explanations for H5N1 pathogenesis concerning its expansive cell and tissues tropism, its promotion of immune dysregulation, and systemic spread. Understanding the mechanism involved in trogocytosis-promoted infection may lead us towards developing new strategies for controlling the spread of this virus and its immunopathology.

## Supporting information

S1 Fig A schematic illustration of the experimental design for Figs 2, 3A, 3B and 4. (TIF)

S2 Fig A schematic illustration of the experimental design for Fig 3C–3E. (TIF)

S3 Fig A schematic illustration of the experimental design for Fig 3F. (TIF)
