# Supplementary figures and images for: Trogocytosis with monocytes associated with increased α2,3 sialic acid expression on B cells during H5N1 influenza virus infection
Source: PLoS One. 2020 Sep 18;15(9):e0239488. doi: 10.1371/journal.pone.0239488 (PMC7500609; doi:10.1371/journal.pone.0239488)

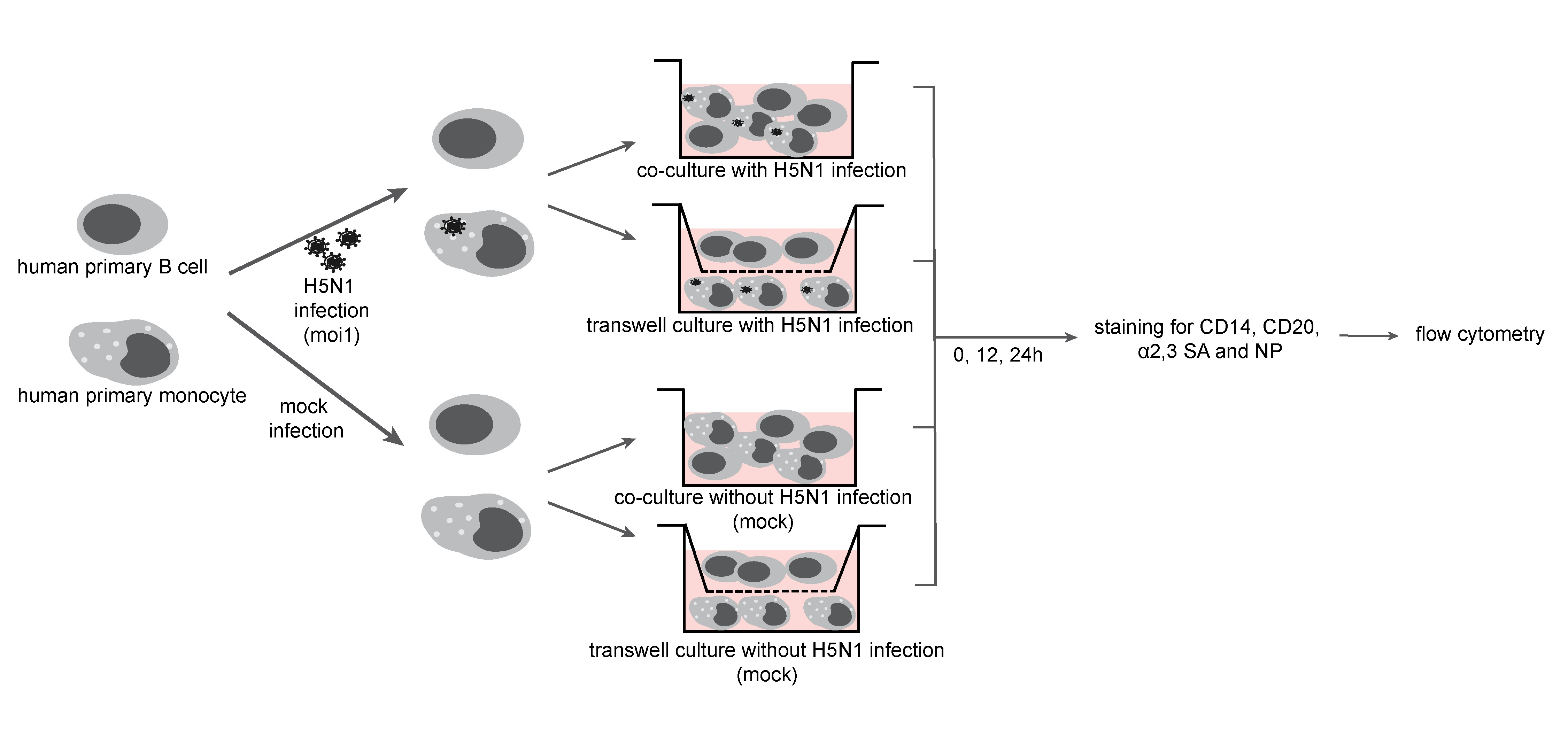

Supplement: S1 Fig — (TIF) [file pone.0239488.s001.tif]

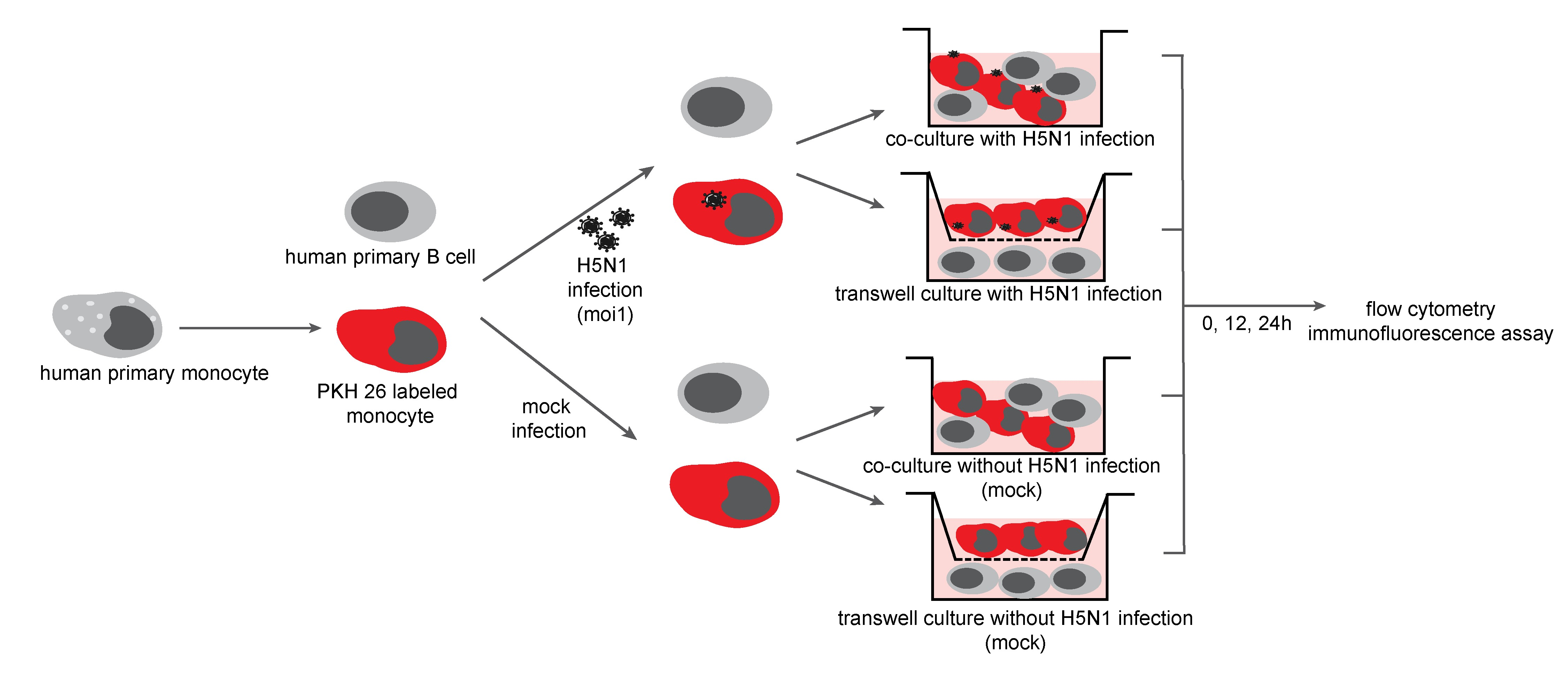

Supplement: S2 Fig — (TIF) [file pone.0239488.s002.tif]

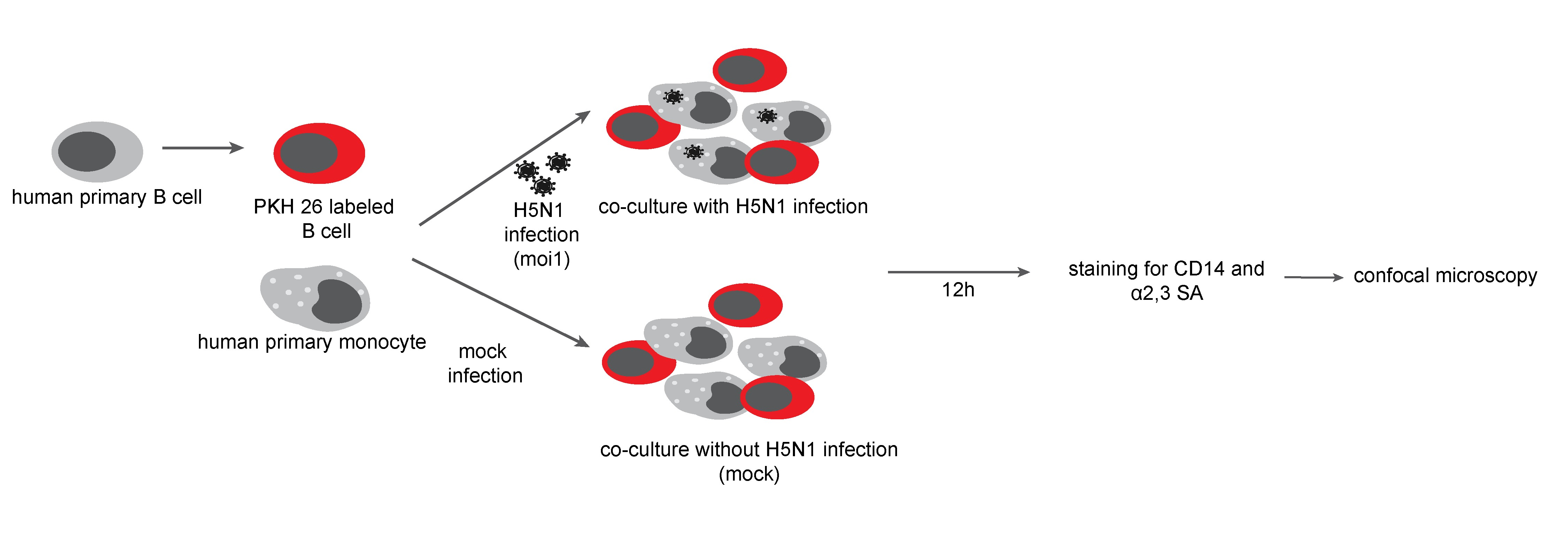

Supplement: S3 Fig — (TIF) [file pone.0239488.s003.tif]
